# Multimodal Retinal Imaging in Asymptomatic APOE ε4 Carriers, APOE ε4 Noncarriers, and Alzheimer's Disease

**DOI:** 10.1002/alz70856_097150

**Published:** 2025-12-24

**Authors:** Alex Choi, Hemal Patel, Sandra Stinnett, Dilraj S Grewal, Sharon Fekrat

**Affiliations:** ^1^ Duke University School of Medicine, Durham, NC, USA; ^2^ Duke University, Durham, NC, USA; ^3^ Duke Eye Center, Durham, NC, USA

## Abstract

**Background:**

We assessed differences in retinal structure and microvasculature among individuals with Alzheimer's disease (AD), APOE ε4 carriers, and APOE ε4 noncarriers.

**Method:**

Participants underwent cognitive evaluation with Mini‐Mental State Examination. Optical coherence tomography (OCT) angiography (Zeiss Cirrus HD‐5000 AngioPlex) vessel density (VD) and perfusion density (PD) in the Early Treatment Diabetic Retinopathy Study 3‐mm and 6‐mm circles and rings were analyzed. OCT angiography peripapillary measurements (capillary perfusion density (CPD), capillary flux index (CFI)) and OCT retinal thickness parameters (retinal nerve fiber layer (RNFL), ganglion cell‐inner plexiform layer (GC‐IPL), central subfield thickness (CST)) were also compared among groups. Participants with diabetes, glaucoma, or vitreoretinal pathology were excluded. Age and sex‐adjusted generalized estimating equations were used for statistical analysis.

**Result:**

275 eyes from 150 AD participants, 373 eyes from 189 cognitively normal APOE ε4 carriers, and 422 eyes from 214 cognitively normal APOE ε4 noncarriers were enrolled. 6mm circle PD and 6mm inner ring VD were significantly lower in APOE ε4 carriers vs noncarriers (*p* = 0.037 and *p* =  0.047, respectively). AD did not have significantly different retinal microvasculature measurements vs APOE ε4 carriers except for peripapillary CPD, which was significantly higher in AD vs APOE ε4 carriers or noncarriers (*p* = 0.003 & *p* < 0.001, respectively). Comparing AD to noncarriers, CST (*p* = 0.014), 6mm circle PD (*p* = 0.025), 6mm circle VD (*p* = 0.036), and 6mm inner ring VD (*p* = 0.042) were significantly lower in AD vs APOE ε4 noncarriers. GC‐IPL thickness, RNFL thickness, 3mm circle and ring VD and PD, and peripapillary CFI did not differ significantly among or between groups (all *p* > 0.05).

**Conclusion:**

APOE ε4 carriers generally did not have significant differences in retinal microvasculature compared to AD, but showed differences compared to ε4 noncarriers, which may indicate that ε4 carriers have begun to show biomarker signs of AD without clinical symptoms. Peripapillary CPD was significantly higher in AD vs APOE ε4 carriers, which may be due to imaging time point during disease course. Longitudinal studies may better elucidate the role of these potential biomarkers along the AD spectrum.

